# Scientific Integrity Principles and Best Practices: Recommendations from a Scientific Integrity Consortium

**DOI:** 10.1007/s11948-019-00094-3

**Published:** 2019-02-27

**Authors:** Alison Kretser, Delia Murphy, Stefano Bertuzzi, Todd Abraham, David B. Allison, Kathryn J. Boor, Johanna Dwyer, Andrea Grantham, Linda J. Harris, Rachelle Hollander, Chavonda Jacobs-Young, Sarah Rovito, Dorothea Vafiadis, Catherine Woteki, Jessica Wyndham, Rickey Yada

**Affiliations:** 1 ILSI North America, Washington, DC USA; 2 American Society for Microbiology, Washington, DC USA; 3 Formerly of the ILSI Global Board of Trustees, Washington, DC USA; 4 Indiana University School of Public Health, Bloomington, IN USA; 5 Cornell University, Ithaca, NY USA; 6 Tufts Medical Center, Boston, MA USA; 7 Canadian Nutrition Society, Ottawa, ON Canada; 8 International Association for Food Protection, Des Moines, IA USA; 9 University of California, Davis, CA USA; 10 Formerly of The National Academies of Sciences, Engineering, and Medicine, The National Academy of Engineering, Center for Engineering Ethics and Society, Washington, DC USA; 11 U.S. Department of Agriculture, Agricultural Research Service, Beltsville, MD USA; 12 Association of Public and Land-Grant Universities, Washington, DC USA; 13 American Heart Association, Washington, DC USA; 14 Department of Food Science and Human Nutrition, Iowa State University (Formerly of the U.S. Department of Agriculture, Research, Education, and Economics), Ames, IA USA; 15 American Association for the Advancement of Science, Washington, DC USA; 16 University of British Columbia, Vancouver, BC Canada; 17 Present Address: Kellan, Washington, DC USA; 18 Present Address: National Council on Aging, Arlington, VA USA

**Keywords:** Culture of integrity, Responsible conduct of research (RCR), Quality of research, Open science, Research misconduct, Detrimental research practices

## Abstract

A Scientific Integrity Consortium developed a set of recommended principles and best practices that can be used broadly across scientific disciplines as a mechanism for consensus on scientific integrity standards and to better equip scientists to operate in a rapidly changing research environment. The two principles that represent the umbrella under which scientific processes should operate are as follows: (1) Foster a culture of integrity in the scientific process. (2) Evidence-based policy interests may have legitimate roles to play in influencing aspects of the research process, but those roles should not interfere with scientific integrity. The nine best practices for instilling scientific integrity in the implementation of these two overarching principles are (1) Require universal training in robust scientific methods, in the use of appropriate experimental design and statistics, and in responsible research practices for scientists at all levels, with the training content regularly updated and presented by qualified scientists. (2) Strengthen scientific integrity oversight and processes throughout the research continuum with a focus on training in ethics and conduct. (3) Encourage reproducibility of research through transparency. (4) Strive to establish open science as the standard operating procedure throughout the scientific enterprise. (5) Develop and implement educational tools to teach communication skills that uphold scientific integrity. (6) Strive to identify ways to further strengthen the peer review process. (7) Encourage scientific journals to publish unanticipated findings that meet standards of quality and scientific integrity. (8) Seek harmonization and implementation among journals of rapid, consistent, and transparent processes for correction and/or retraction of published papers. (9) Design rigorous and comprehensive evaluation criteria that recognize and reward the highest standards of integrity in scientific research.

## Introduction

In the twenty-first century, scientists work in a research environment “that is being transformed by globalization, interdisciplinary research projects, team science, and information technologies” (Interacademy Partnership 2016). As the scientific enterprise evolves, all stakeholders in the scientific community have an ethical obligation to place a high priority on instilling and championing the highest standards of scientific integrity in these new settings and applications.

The North American Branch of the International Life Sciences Institute (ILSI North America) and the U.S. National Academies of Sciences, Engineering, and Medicine’s Government-University-Industry Research Roundtable (GUIRR) share a commitment to upholding scientific integrity and therefore organized a group that also shares these values. In early 2017, ILSI North America convened a meeting of the Scientific Integrity Consortium (“the Consortium”), hosted by GUIRR at the National Academy of Sciences building in Washington, DC. The Consortium, which includes representatives from four U.S. government agencies, three Canadian government agencies, eleven professional societies, six universities, and three nonprofit scientific organizations, came together to develop a set of principles and best practices for scientific integrity that can be used broadly across all scientific disciplines. The goal of the Consortium was to have broad representation from multiple scientific disciplines and fields. Most Consortium participants were invited based on their role in their organization and their responsibilities around scientific integrity. The Consortium members were a mix of administrators, scientific integrity officers, deans, executive directors, presidents of professional scientific societies, industry executives, and scientists. Each participant contributed a different perspective to the discussions, which was shaped predominantly by the sector where the individual was employed. Members of the Consortium concluded that while their institutions and organizations may differ in the strategies they use to promote scientific integrity and the extent that they implement them, there must be consensus and alignment around the necessity for scientific integrity standards and their content.

This article describes the recommended two overarching principles and nine best practices for fostering scientific integrity that are particularly salient in the current and emerging context for scientific research, and summarizes the discussion leading to their development.

## Context for Discussion and Definitions

Prior to the Consortium meeting, a draft set of principles was distributed to the participants to begin the discussion. These draft principles were developed using the six recommendations identified in the American Society of Microbiology’s “A Framework for Improving the Quality of Research in the Biological Sciences” (Casadevall et al. 2016) and five additional principles that emerged from the ILSI North America publication “Scientific Integrity Resource Guide: Efforts by Federal Agencies, Foundations, Nonprofit Organizations, Professional Societies, and Academia in the United States” (Kretser et al. 2017). The Consortium used these draft principles as the basis of the discussion and reconstructed them to form the final set of recommended principles and best practices for scientific integrity.

Scientific integrity depends on a set of foundational expectations that all science should be built upon to maintain trust. Consortium members recognized that work on scientific integrity policies has proceeded for several decades and yet the scientific community continues to experience periodic lapses in this area. Failures of scientific integrity may not be more common now than in the past, but they may be more visible. This is in part due to the widespread availability of electronic publication, which enhances the ease of discovering breaches in scientific integrity, and social media, which makes the public aware of them. The emergence of these tools allows for rapid dissemination of concerns regarding published scientific work. This highlights the timely, indeed urgent need to refocus the scientific community’s efforts on policing itself. The objective in developing this set of principles and best practices is to build upon and advance the extensive work already done by many of the Consortium participants and other organizations to better achieve and uphold scientific integrity. The Consortium members hope that this effort will be embraced and further refined by the global scientific community.

There is currently no universal definition of scientific integrity. Yet an agreed-upon definition of scientific integrity and other key terms is crucial to understanding the boundaries of the principles and best practices, The Institute for Defense Analyses (IDA) *Review of Federal Agency Policies on Scientific Integrity* found that U.S. federal agency policies vary in their approach to defining scientific integrity (Nek and Eisenstadt 2016). Some agencies’ definitions incorporate research integrity as part of scientific integrity, while other agencies separate both terms. The Consortium agreed that for the purposes of these principles and best practices, the terms “scientific integrity” [as defined by the U.S. Department of the Interior (DOI)], “research misconduct” (as defined by the U.S. Federal Research Misconduct Policy), and “detrimental research practices” (as defined by the U.S. National Academies of Sciences, Engineering, and Medicine 2017 report *Fostering Integrity in Research*) would be used in the development of the principles and best practices for scientific integrity. These definitions are as follows:

- *Scientific integrity* The DOI developed a definition of scientific integrity that was then adopted in various forms by five other federal agencies. The DOI defines scientific integrity as “the condition that occurs when persons… adhere to accepted standards, professional values, and practices of the relevant scientific community… Adherence to these standards ensures objectivity, clarity, and reproducibility, and utility of scientific and scholarly activities and assessments and helps prevent bias, fabrication, falsification, plagiarism, outside interference, censorship and inadequate procedural and information security…” (Nek and Eisenstadt 2016, p. 11).
- *Research misconduct* The Federal Research Misconduct Policy sets forth a uniform definition of research misconduct, defined as “fabrication, falsification, or plagiarism in proposing, performing or reviewing research, or in reporting research results. Research misconduct does not include honest error or differences of opinion” (Nek and Eisenstadt 2016, p. 13). Canadian federal research agencies use the term “breach” rather than “research misconduct,” in which “breach” is defined as the “failure to comply with any Agency policy throughout the life cycle of a research project—from application for funding, to the conduct of the research and the dissemination of research results” (Canadian Institutes of Health Research et al. 2016). Breaches include fabrication, falsification, destruction of research records, plagiarism, redundant publication or self-plagiarism, invalid authorship, inadequate acknowledgement, mismanagement of conflict of interest, misrepresentation in a grant application or related documents, mismanagement of grants or award funds, breach of agency policies or requirements for certain types of research, and breach of agency peer review processes (Canadian Institutes of Health Research et al. 2016).
- *Detrimental research practices* The report, *Fostering Integrity in Research*, coined a new term, “detrimental research practice,” for instances when researchers “engage in other behavior [beyond research misconduct] that clearly damages research” (National Academies of Sciences, Engineering, and Medicine 2017, p. 1). When researchers commit detrimental research practices or research misconduct, they “stray from the norms and appropriate practices of science” (National Academies of Sciences, Engineering, and Medicine 2017, p. 1). The Consortium supports the use of this new term. A similar term, “questionable research practices” (QRP) (John et al. 2012), is used in Canada and abroad.

The Consortium also considered the context or “ecosystem” of scientific integrity in the research environment and how “responsibility for ensuring integrity is borne by many different people and organizations, starting with individual researchers but including research supervisors and funders, institutional leaders, peer reviewers and journal editors. The analogy of a ‘research environment’ [as an ecosystem] is an apt one—this is a complex ecosystem, and therefore, attention must be given not only to individual behavior, such as research misconduct, but also to the systems that affect it, such as academic rewards, incentives and pressures” (Wager 2015). All the ecosystem components and players must act synergistically and in a trustworthy way for science to see continuous improvements in its output. The Consortium set out to identify what can compromise this ecosystem and erode the scientific process and considered how to incorporate these concepts into the principles and best practices.

## Principles and Best Practices for Scientific Integrity

The Consortium developed two overarching principles that represent the umbrella under which scientific processes should operate and nine best practices for instilling scientific integrity through the implementation of the two overarching principles (Box 1).

**Box 1:** Principles and best practices for scientific integrity

Overarching principles for fostering scientific integrity 1. Foster a culture of integrity in the scientific process 2. Evidence-based policy interests may have legitimate roles to play in influencing aspects of the research process, but those roles should not interfere with scientific integrity Best practices for fostering scientific integrity 1. Require universal training in robust scientific methods, in the use of appropriate experimental design and statistics, and in responsible research practices for scientists at all levels, with the training content regularly updated and presented by qualified scientists 2. Strengthen scientific integrity oversight and processes throughout the research continuum with a focus on training in ethics and conduct 3. Encourage reproducibility of research through transparency 4. Strive to establish open science as the standard operating procedure throughout the scientific enterprise 5. Develop and implement educational tools to teach communication skills that uphold scientific integrity 6. Strive to identify ways to further strengthen the peer review process 7. Encourage scientific journals to publish unanticipated findings that meet standards of quality and scientific integrity 8. Seek harmonization and implementation among journals of rapid, consistent, and transparent processes for correction and/or retraction of published papers 9. Design rigorous and comprehensive evaluation criteria that recognize and reward the highest standards of integrity in scientific research	

### Overarching Principles for Fostering Scientific Integrity

1. Foster a culture of integrity in the scientific process

The Consortium agreed unanimously that it is paramount to create a culture of integrity in science that rewards scientific integrity and professional excellence, fosters an environment in which open discussion reflects a balance of diverse scientific views, and is committed to transparency, honesty, and thorough consideration of research outcomes. To create a culture of integrity, significant systemic, organizational, and psychological changes in the global research culture will need to be made. For example, a five-part Lancet (2014) series on “Research: Increasing Value, Reducing Waste” laid out pressing issues and recommendations for how to increase value and reduce waste in biomedical research and provided seventeen recommendations that are addressed to five main stakeholders (funders, regulators, journals, academic institutions, and researchers). The implementation of these recommendations by the five stakeholder groups was then mapped out in an article, “Increasing Value and Reducing Waste in Biomedical Research: Who’s Listening” (Moher et al. 2016), which also provided recommendations for ways to ensure further adoption of the *Lancet* series recommendations.

It is imperative that preconceived notions about scientific integrity and conduct be addressed when considering how to instill a culture of scientific integrity. Mary L. Devereaux (2014) identified four barriers to scientists’ thinking about the social and ethical implications of their work: (1) an absence of awareness; (2) not seeing the connection between scientific work and broader social issues; (3) overconfidence in the ability to handle ethical problems “on the spot”; and (4) the ethical dimensions of research being seen as at odds with “the daily practice of science” (Devereaux 2014, p. 166). These barriers were discussed by the Consortium as ongoing issues in the scientific community and were considered during the development of the principles and best practices. To combat these preconceived barriers, institutions should consider developing or enrolling researchers in programs similar to the P.I. [Principal Investigator] Program for professionalism and integrity in research at Washington University in St. Louis (2018a), which offers personalized assessments, group workshops, and postworkshop coaching calls to help researchers operate professionally in today’s complex environments. The P.I. Program’s approach could be one way that an institution could reimagine its support infrastructure to maintain scientific integrity.

A culture of scientific integrity is affected by the different generations of scientists in the workforce. For example, the initial training a scientist received during his or her schooling and early years may not have provided the knowledge needed to navigate the current scientific research environment and expected standards with respect to “p-hacking”^1^ (Head et al. 2015) and other detrimental research practices. Technological advancements, global collaborations, multidisciplinary teams, and other factors have had an enormous impact on the research environment, so norms of behavior for scientists are not the same as they were even 10 years ago. With these advances, the expectations for integrity in science have become more complex.

Additionally, there are different obstacles throughout the various stages of a scientist’s career that may challenge adherence to scientific integrity or encourage a scientist to cut corners. Scientists may find it challenging to secure funding for their research and therefore may engage in practices inconsistent with scientific integrity. Some scientists may resist change or may feel that they do not need to follow consensus-based guidelines given their expertise and experience. Continuing education and training may help to negate these challenges and are crucial to keep scientific integrity top of mind.

Part of the existing reward system of promotions and tenure, salaries, book deals, speaking invitations, and so forth is tied to publishing in high-profile journals and acquiring grants, perpetuating the “publish or perish” mentality and raising other difficulties in instilling a culture of scientific integrity. Outcomes are often tied to reporting seemingly “exciting” findings more than rigorously produced findings. According to Devereaux (2014), “in a well-functioning profession, the reward systems and normative ideals align. The real threat to ethical conduct in science lies here—in the tension between the existing reward systems and the norms of science” (Devereaux 2014, p. 168). “Institutions must support and reward researchers who do solid—not just flashy—science and hold to account those whose methods are questionable” (Begley et al. 2015). Suggestions to address this necessary change are found later in Best Practices 4, 6, 7, and 9.

To nurture a culture of integrity, institutions must develop policies, procedures, and practices that address scientific integrity, provide training of personnel, and work continuously to maintain awareness and advocacy for these practices. While the role of the institution in fostering a culture of integrity is often focused on a compliance-centered and training-based approach, it is equally important for an institution to implement a supportive approach that helps everyone meet pre-established expectations. Institutions should re-consider their central support infrastructure and how it facilitates research accountability. For example, the lack of funding for electronic notebooks, lack of research quality management and quality assurance support, and lack of equipment calibration strategies fail to establish the optimal environment that allows scientists to operate with scientific integrity. Similar to the Consortium’s recommendation, Begley and colleagues (2015) suggest developing a system for research they call good institutional practice (GIP), which should include six tenets, including routine discussion of research methods and records and quality management, that provide specific encouragement for institutions to support scientists in their effort to conduct sound science beyond training programs and compliance strategies.

The IDA *Review of Federal Agency Policies on Scientific Integrity* provides recommendations for ways to promote a culture of scientific integrity, including the following:

1. Provide an agency-specific context for why scientific integrity is important to an agency’s mission and activities.
2. Train scientists and nonscientists on the importance of scientific integrity.
3. Provide a process for resolving differences in scientific opinions.
4. Issue periodic bulletins or newsletters to remind personnel of the importance of scientific integrity (Nek and Eisenstadt 2016, p. vi).

The Consortium agrees that the IDA recommendations, when implemented, can help to create a culture of scientific integrity that is pervasive throughout institutions in the scientific community and can be an effective change agent. For example, in reference to the second IDA recommendation, many government agencies now require scientific integrity training for employees and others associated with their agencies, including recipients of grants and contracts. The U.S. National Institutes of Health (NIH) requires all recipients of NIH Institutional Research Training Grants, Individual Fellowship Awards, Career Development Awards (Institutional and Individual), Research Education Grants, Dissertation Research Grants, or other grant programs to be instructed in the responsible conduct of research (RCR) (National Institutes of Health 2011). The National Science Foundation (2017) and the U.S. Department of Agriculture (USDA) National Institute of Food and Agriculture (2013, p. 5) also require training in RCR for grant recipients. In Canada, the Panel on Research Ethics (2017) developed an online tutorial course on research ethics based on the “Tri-Council Policy Statement: Ethical Conduct for Research Involving Humans.” Many Canadian institutions use this educational resource, and some institutions have made the tutorial a requirement for their research ethics boards, students, and faculty. Surveys could also be used to serve as a periodic reminder of scientific integrity. Beyond these examples from federal agencies, professional societies, academic institutions, and other organizations all have a responsibility to keep scientific integrity visible through these same methods as well as through the development of training modules.

The Consortium believes it is time for a standardized approach to research conduct, which will help to re-establish and strengthen trust in research and in the scientific community. The case was made by the Global Biological Standards Institute (GBSI) in 2013 for the development and use of standards in life science research to improve its credibility, reproducibility, and translatability. GBSI has focused their efforts on the lack of standards in four areas: basic biologic research, cell line misidentification, research antibodies, and propagation of high-throughput technologies (HTS) artifacts (Freedman and Inglese 2014) and has evaluated their progress since 2013 in a recent publication (Freedman et al. 2017). Standards in the area of preclinical science have been the focus of the European College of Neuropsychopharmacology (ECNP) (2018), whose network has been working to systematically advance the status of preclinical research through identifying best research practices, developing and implementing novel data quality standards, and providing recommendations to the neuroscience community. The European Quality in Preclinical Data (EQIPD) project is also working to establish, evaluate, and provide training in principles and practices associated with research rigor (Innovative Medicines Initiative 2018).

*Consortium recommendation* To ensure the trust of the scientific community and public at large in study results, it may be helpful to develop a broad *checklist* that incorporates a set of standard procedures or best practices for scientific integrity. This checklist could be used by scientists in laboratories, for research studies, or in the development of publications. It could serve as both a guide to the research design, conduct, and reporting of studies and also as an objective tool for the evaluation of published research, although it is recognized that different fields of science may require adjustments or additions to a standard checklist. Some of the principles and best practices described here, as well as the comprehensive Reproducibility 2020 action plan from GBSI (Freedman et al. 2017) and other resources, could be the foundation for the development of this checklist.

The checklist could be a partial basis for a set of criteria for a “stamp of approval” or “accreditation badges” that could be visible on a laboratory’s website, on a data set, or on a publication, showing which scientific integrity practices were followed. These badges could follow the example of those developed by the Center for Open Science. The Center for Open Science (2017b) states that these “badges are included on publications and signal to the reader that the content of the publication has been made publicly available and certify its accessibility in a persistent location. They acknowledge open science practices are incentives for researchers to share data, materials, or to preregister protocols and have proven to be successful and continue to gain visibility in the scientific community.” A recent systematic review identified the Center for Open Science’s badging program as the only evidence-based incentive program that was effective at increasing the rates of data sharing (Rowhani-Farid et al. 2017). This is encouraging because it shows that the concept of badges or a stamp of approval could be useful in other areas of scientific integrity as well.

2. Evidence-based policy interests may have legitimate roles to play in influencing aspects of the research process, but those roles should not interfere with scientific integrity

This principle addresses the interface of science and policy. Most scientific research is carried out with the goal of producing information that will be useful to society, whether to further future scientific research and discovery or as applied to address immediate societal needs. One important practical application of scientific information is its use as evidence to inform policy decisions. Science must play a central role in the formulation of evidence-based policy making.

Regulatory agencies have the responsibility to use scientific evidence to implement laws and develop regulations. However, the “production of evidence itself is not value-free, and … inherent biases and limitations result from how we frame questions and seek knowledge in the first place” (European Commission 2015). When outcomes of research are used to address disputed policy issues, a conscious, disciplined commitment to scientific integrity is critical, especially in the study design and the translation and communication of results, which may be influenced by this awareness.

It is impossible to eliminate all the subjective factors that may subtly influence how individuals think about and approach the formulation and solving of problems. Therefore, it is all the more important that established research procedures be scrupulously followed, that study limitations be acknowledged, and that the data on which results are based be available to the maximum extent allowed by good research practice to assist in review and evaluation.

The Consortium agreed that the interests and priorities of policy makers sometimes affect the questions asked by scientists; however, the ultimate use of science in public policy, as well as decision making and public opinion, should not affect the content of the science.

### Best Practices for Fostering Scientific Integrity

1. Require universal training in robust scientific methods, in the use of appropriate experimental design and statistics, and in responsible research practices for scientists at all levels, with the training content regularly updated and presented by qualified scientists

Rigorous implementation of the scientific method (Merriam Webster 2018) helps ensure the integrity of research. According to Casadevall and colleagues (2016), “Given that the quality of a scientist’s output is often a reflection of his/her training, one obvious mechanism to improve the quality of [research] is to improve the training of scientists.” A scientist must be grounded in the basic principles of robust scientific methods to achieve and maintain scientific integrity within the growing complexity of the research environment. For example, at the U.S. Environmental Protection Agency (EPA), every laboratory must go through research integrity accreditation programs. New employees are required to watch videos on scientific integrity so that they understand how scientific integrity enhances their work. Additionally, the EPA intends that all employees periodically complete a questionnaire on scientific integrity, which is a reminder of the responsibilities of doing work with integrity (Scientific Integrity Consortium meeting discussion, 2 March 2017).

While work to improve training in this area has been done, many scientists still do not receive sufficient training of this sort. Casadevall and colleagues (2016) state that “Training in ethics and the responsible conduct of science is already a common feature of scientific training programs. However, it is often seen more as a rite of passage to be completed in the quest for a scientific degree than as an integral component of a system that seeks to improve the quality of science.” University leaders should better promote the critical importance of research quality (Schrag and Purdy 2017), institutions must work to continuously update this type of training, and scientists should be required to repeat this training periodically across their career. Common unintentional errors and habits that could lead to detrimental research practices need to be highlighted as part of trainings, so scientists can recognize them in their own research. A scientific field that polices itself is key to maintaining scientific integrity.

Professional societies and foundations have a critical role to play in developing the training standards pertinent to their fields. Many professional societies’ accreditation programs require training in scientific integrity, and some already offer training on an ongoing basis or at their annual meetings to share good practices, challenges, and solutions in implementing scientific integrity policies. These types of programs should be encouraged and become more widespread. These programs also need to be studied to determine how helpful they are and how they can be improved.

NIH defines RCR as “the practice of scientific investigation with integrity. It involves the awareness and application of established professional norms and ethical principles in the performance of all activities related to scientific research (National Institutes of Health 2011).” RCR training should include education on the responsibilities expected of researchers and scientists, the types of research misconduct and detrimental research practices that can arise when deviating from these responsibilities, and the potential consequences of deviating. The U.S. Department of Health and Human Services Office of Research Integrity (HHS ORI 2017) has a list of “Case Studies of Misconduct” that can be used as part of an educational program on consequences of research misconduct. Similarly, in Canada, the Secretariat on Responsible Conduct of Research developed RCR file summaries of confirmed breaches that can be used as an educational tool for institutional RCR education programs (Government of Canada 2017). There are also video games and interactive websites that help to train scientists in ethical behavior. However, there can sometimes be a disconnect between those teaching RCR courses and the scientists receiving the training, as the RCR courses are often taught by administrators or individuals who are not scientists. The Consortium members suggested that in order for RCR courses to be more impactful, they should be taught by scientists—ideally by those who are knowledgeable in the rapid advances in technology relevant to the scientific field.

Mentorship is vital in the scientific community. HHS ORI has found that the majority of research misconduct cases include deficiencies in the mentorship of the individual who committed the misconduct (Tamot and Hammatt 2017; Wright et al. 2008). Training in science often follows an apprenticeship model, so training in good mentorship for those in mentor positions should be developed and required. One such program is the Delta Program for Research Mentor Training at the University of Wisconsin–Madison (2018). Completing this type of training would improve the skills of the mentor and could plausibly decrease detrimental research practices and research misconduct.

Complications can arise in developing scientific integrity training for many emerging fields of scientific study. There are fields in which newly developed tools and techniques are allowing scientists to generate large volumes of data, but the lack of validation leads to varied interpretations of these new types of data and is fraught with uncertainties.

Appropriate statistical analyses are just as important to scientific integrity as how the data were collected or generated. The design of a study affects the type of statistical analysis that can be applied to the data generated by the study (National Academy of Sciences 2017). Inclusion of statisticians from the onset of studies across different disciplines, meaning that they are collaborating right from the point of experimental design, will enhance the rigor of the resulting research.

2. Strengthen scientific integrity oversight and processes throughout the research continuum with a focus on training in ethics and conduct

While the first best practice focuses mainly on the training of individuals in the scientific community, this one urges institutions to strengthen their scientific integrity oversight. According to the National Academy of Sciences, Engineering, and Medicine (2017), “Addressing threats to scientific integrity requires a contemporary understanding of the research system and challenges to the integrity of that system” (p. 1). It is incumbent upon institutions, as part of their responsibility to foster a culture of scientific integrity, to establish comprehensive, consistent, and transparent systems to detect and report problems to both their own research institutions and other entities as required, such as HHS ORI (Davies et al. 2016, p. 10).

Strengthening scientific integrity oversight and processes must begin at the highest level of an organization, although it is ultimately the responsibility of all researchers in an institution to maintain the integrity of research. There needs to be a commitment to recognizing scientific integrity as an integral part of the values of the research enterprise, and this should begin with an institutional shift from encouraging training in scientific integrity to making it mandatory and expressly integrating the principles of scientific integrity into all relevant policies, processes, and practices of the institution.

The processes for handling allegations vary among institutions. Ultimately, it is important for all institutions to have a scientific integrity policy that researchers facing such issues can reference that includes a process for adjudicating instances of irresponsible research when suspicion of detrimental research practices and research misconduct arises. The initial effort to establish processes for responding to loss of integrity is considerable, but establishing these processes ahead of time pays off in two ways: (1) the institution is better equipped to prevent, or at least reduce, instances of detrimental research practices or research misconduct from occurring; and (2) a system is in place to deal with allegations when they arise, including the treatment of whistleblowers, agreed-upon proportionate penalties for instances of confirmed research misconduct and detrimental research practices, and processes for the correction of the research record. By doing this, an institution puts itself in the desirable position of being proactive, rather than reactive. Additionally, the Consortium discussed how it is often the case that the individual within an institution that has responsibility for oversight of scientific integrity may or may not have a science background, and he or she may have a different position depending on the institution (i.e., the individual could work in the President’s office or in a different division in an agency, etc.). This can impact what an institution focuses on more within its processes or how policies are implemented.

U.S. federal agencies have focused on the development of scientific integrity policies as part of their responses to the 2009 Presidential Memorandum on Scientific Integrity (The White House 2009). Consortium participants representing different federal agencies have been gratified by the development and implementation of these policies within their agencies. Several of these agencies have had allegations of detrimental research practices and research misconduct and found they were well equipped to address them when they arose. Canadian federal research agencies are also well equipped to handle allegations because of the requirements for Canadian institutions to have a research integrity or RCR policy that meets the minimum requirements of the “Tri-Agency Framework: Responsible Conduct of Research” (Canadian Institutes of Health Research et al. 2016).

Other Consortium members acknowledged that their institutions still had work to do to reach this same level of preparedness. The use of a checklist like the one proposed by an expert group for research integrity investigation will help standardize investigations into allegations of research misconduct and detrimental research practices. The checklist is “designed to address whether an investigation follows reasonable standards and if the subsequent report is appropriate and complete” (Gunsalus et al. 2018).

The 2017 report, *Fostering Integrity in Research*, includes eleven recommendations for fostering integrity in research. One of the recommendations calls for the establishment of a Research Integrity Advisory Board (RIAB), which would be established as an independent nonprofit organization. The RIAB “will work with all stakeholders in the research enterprise—researchers, research institutions, research sponsors and regulators, journals, and scientific societies—to share expertise and approaches for addressing and minimizing research misconduct and detrimental research practices” (National Academies of Sciences, Engineering, and Medicine 2017, p. 5). Although there are other institutions that are already doing these things, “none has research integrity as its sole focus nor covers so much territory” (Mervis 2017). The establishment of a RIAB would be immensely helpful to institutions that are working to improve oversight and processes in scientific integrity and can use the resources of the RIAB. By providing standardized materials, the RIAB could provide a means for rapid dissemination of best practices, such as the work of the Consortium. Further, the RIAB could assist in propagation of training programs that promote scientific integrity, such as the P.I. Program at Washington University in St. Louis (2018b) or the Delta Program for Research Mentor Training at the University of Wisconsin–Madison (2018). The role of the proposed RIAB would be similar to the role of the Secretariat on Responsible Conduct of Research in Canada (2015).

The 21st Century Cures Act (2016) includes a directive for the establishment of a Research Policy Board whose purpose and responsibilities include ensuring that regulations are consistent with maintaining responsible oversight of federally funded research in the prevention of detrimental research practices and research misconduct. Section 2034(f)(3) states that the Research Policy Board also has responsibility for ensuring that scientific integrity is not compromised by challenges emerging from new scientific advances. Once established, the Research Policy Board would benefit from the work of the RIAB. Section 2039(a) of the Act also authorizes the Secretary of Health and Human Services, acting through the Director of the NIH, to convene an Advisory Committee to issue recommendations to enhance the rigor and reproducibility of scientific research.

3. Encourage reproducibility of research through transparency

Transparency in reporting is both an ethical responsibility and a scientific obligation. Scientific knowledge is dependent on reproducibility of research results, which cannot be assured if the methods and the data are not adequately available. Therefore, it is incumbent upon the scientific community to support an ecosystem that encourages scientists to enhance reproducibility through transparency of their work. Some institutions have begun to examine and adopt mechanisms which encourage transparency. Examples include the (1) Agency for Healthcare Research and Quality (2017), which maintains a public systematic review database repository; (2) the Federation of American Societies for Experimental Biology (2016), which published a set of recommendations in 2016 on enhancing reproducibility in research utilizing mouse models or antibodies; and (3) the Center for Open Science (2017a), whose mission is to increase openness, integrity, and reproducibility of research through its programs, including the Open Science Framework and the TOP Guidelines. Munafò and colleagues (2017) offer a series of measures, which they have organized into categories (methods, reporting and dissemination, reproducibility, evaluation, and incentives), that they believe will improve research efficiency and robustness of scientific findings by directly targeting specific threats to reproducible science. The categories are intended to provide an evidence-based set of actions that can be implemented by researchers, institutions, journals, and funders (Munafò et al. 2017).

To enhance reproducibility, some scientific journals are encouraging “the use of checklists for authors of submitted papers to assess the rigor of experimental design. *Nature* journals now require the submission of a reporting checklist for Life Science Articles to provide details on experimental design and statistics, biological reagent validation, and data sharing. NIH guidelines for reporting preclinical research also encourage the development of best practice guidelines for digital data and validation of biological reagents. Many journals and societies have endorsed the NIH guidelines, which should lead to continued adaptation of journal policy to NIH guidelines” (Davies et al. 2016, p. 11).

While an emphasis on encouraging reproducibility is building in the scientific community, questions still remain: Are these problems of rigor and reproducibility occurring more frequently in some fields of science than in others? Are there certain areas to focus on? Is there a particular problem that has arisen in certain fields of science that can be learned from and applied to other fields? The 2017 Sackler Colloquium on Reproducibility of Research Issues and Proposed Remedies examined these types of questions. The colloquium brought together scientists and researchers from multiple disciplines to determine the scope of the problems of reproducibility in a more tactical way that permits each problematic aspect to be evaluated, measured, assessed for baseline levels, targeted with proposed interventions to reduce occurrences, and monitored for improvement (National Academy of Sciences 2017). Authors of the various proceedings from the Sackler Colloquium collectively made some of the following points: (1) Breaches in research rigor, reproducibility, and transparency and research errors clearly do occur with sufficient frequency to be notable. (2) However, for most aspects, the exact frequency is unknown. (3) Whether the relative frequency of such breaches and errors has been increasing, decreasing, or remaining constant over the years is largely unknown. (4) Efforts to reduce such breaches and errors are warranted and specific techniques ranging from regulations to infrastructure support to investigator training are all warranted and currently being expanded (Allison et al. 2018). All of these points were discussed by the Consortium, in particular the final point. The Consortium too felt strongly that it was important for the scientific community to acknowledge the existence of these breaches and errors and focus on applying these types of methods to help reduce them. The principles and best practices put forward here provide a framework to accomplish this goal.

Aspects of this best practice are interrelated with open science and can be implemented by the recommendations in Best Practice 4.

4. Strive to establish open science as the standard operating procedure throughout the scientific enterprise

Open science is “the movement to make scientific research, data and dissemination accessible to all levels of an inquiring society, amateur or professional. It encompasses practices such as publishing open research, campaigning for open access, encouraging scientists to practice open notebook science, and generally making it easier to publish and communicate scientific knowledge” (Wikipedia 2017b). The FOSTER Consortium (2017) defines open science as “the practice of science in such a way that others can collaborate and contribute… under terms that enable reuse, redistribution and reproduction of the research and its underlying data and methods.”

Many institutions have made strides in recent years to develop and adopt open science policies, data access plans, and tools, and they are beginning to implement requirements for transparency and for supporting reproducibility. For example, the NIH has data sharing policies that apply to broad sets of investigators and data, as well as individual requests for applications and program announcements that may specify additional requirements or expectations for data sharing that apply to specific projects (National Library of Medicine 2014). The USDA has created Ag Data Commons, a data access system that holds data files managed directly by the USDA National Agriculture Library and links to 250 datasets and resources located on other websites (U.S. Department of Agriculture 2017). The EPA has developed an open data policy implementation plan that includes a component that promotes the importance of efficient release and management of data as an asset (U.S. Environmental Protection Agency 2015). The Canadian Institutes of Health Research, the Natural Sciences and Engineering Research Council of Canada, and the Social Sciences and Humanities Research Council of Canada have implemented the Tri-Agency Open Access Policy on Publications.

As publicly funded organizations, the [Canadian] Agencies have a fundamental interest in promoting the availability of findings that result from the research they fund, including research publications and data, to the widest possible audience, and at the earliest possible opportunity. Societal advancement is made possible through widespread and barrier-free access to cutting-edge research and knowledge, enabling researchers, scholars, clinicians, policy makers, private sector and not-for-profit organizations and the public to use and build on this knowledge… As research and scholarship become increasingly multidisciplinary and collaborative, both domestically and internationally, the Agencies are working to facilitate research partnerships by harmonizing domestic policies and aligning with the global movement to open access (Government of Canada 2016).

The Center for Open Science (2017c) Open Science Framework (OSF) provides free and open source project management support for researchers across the entire research lifecycle. The OSF is a collaboration tool that encourages transparency in both the public and private sectors and helps researchers work on projects privately with a limited number of collaborators and make parts or all of their projects public. As a flexible repository, it can store and archive research data, protocols, and materials (Center for Open Science 2017c).

There are many benefits of data sharing, including (1) to ensure rigor, reproducibility, and integrity; (2) to use as a resource for further research and analysis in order to expand the evidence base; and (3) to encourage public trust. Full transparency in reporting of scientific findings is crucial to ensuring scientific integrity, including the willingness to disclose all findings, whether they support the research hypothesis or not. Yet there are impediments and disadvantages of open science that must be acknowledged, including concerns with intellectual property, matters of national security, and the potential loss of confidentiality of research participants in human clinical trials. For example, if clinical trial participants believe that there is a possibility that personal information will be openly shared, even in anonymized form, then the level of participation of future human subject research could be adversely affected. There are ways to further anonymize data (sets) but implementing them will come at an additional cost, in terms of training in their use and direct utilization as well as financial costs. Misuse of data is also a problem, especially when subjects have not agreed to widespread dissemination of information. This raises ethical issues about informed consent.

Open science is not a trivial requirement and it is important to document the challenges of moving toward this goal. There are different ways to implement this best practice, such as a phased approach to open data or prioritizing open data of newly published research. Significant time and financial resources are required to provide and compile open data. The *Fostering Integrity in Research* report recommends that “U.S. Federal funding agencies and other research sponsors should allocate sufficient funds to enable the long-term storage, archiving, and access of datasets and code necessary for the replication of published findings” (National Academies of Sciences, Engineering, and Medicine 2017, p. 6). It is also important that professional reviews include recognition and incentives for researchers for making data transparent.

Scientists should strive to make open science the norm in the research community. Ultimately, it is in the best interest of all sectors, public and private, that open science becomes the standard operating procedure for transparency. The key is not only in developing open science policies but also in ensuring their execution.

5. Develop and implement educational tools to teach communication skills that uphold scientific integrity

Scientific integrity is essential in the communication of research study findings. Although it is often difficult to communicate results effectively to the various sectors (e.g., the scientific community, policy officials, the media, and the public), scientists should be encouraged to communicate their research findings. Science communication training should teach scientists how to accurately answer questions about the meaning, importance, and limitations of their work, while still maintaining the integrity of the work. Scientists also must be able to discuss and demonstrate the quality of their work with the public, so they can show how they address and ensure research rigor. Development of tools for demonstrating the quality of their work (e.g., badges, certifications, and data repositories) would be helpful. Institutions have the responsibility of requiring ethical science communication training to equip scientists with the tools that permit them to communicate effectively. Effective communication training should be built into institutional training programs discussed in Best Practice 1.

The communications departments or press offices in institutions have a key role in disseminating information about investigators’ research. Thus, it is important to have open lines of communication between scientists and their communications department or press office. Ideally, scientists should help develop and review in advance any communications about their work to ensure accurate context and reflection of their findings.

Communication of research results has become even more complex in the age of social media. In some cases, valid scientific findings are challenged in the media or elsewhere by those who disagree with the conclusions, scientists are accused of suppressing scientific findings, or critics make ad hominem attacks on the scientists themselves rather than offering critiques of studies. While some aspects of social media’s influence on the communication of science can be considered negative, social media may have a positive effect by exposing and deterring detrimental research practices and research misconduct. Anonymous online platforms such as PubPeer also serve as a deterrent, as one of its functions is reporting and commenting on suspected cases of poor practice (Davies et al. 2016, p. 10).

Scientists have an obligation to be accurate and honest in their communications. Approaches that increase accuracy and honesty and reduce spin, obfuscation, and exaggeration are merited.

6. Strive to identify ways to further strengthen the peer review process

The rigor and transparency of the peer review process is vital to scientific integrity. While the journal and its peer reviewers play a role in reviewing the research and outcomes put forth in a manuscript, the authors have the responsibility to verify the authenticity of their work. It is unrealistic to assume that peer review can be the sole gatekeeper of scientific integrity.

Journals currently have varying peer review processes. Making these processes more transparent may ultimately lead to the development of a set of common standards for peer review. The checklist described in principle 1 (once developed) is recommended as the basis for a standardized form for authors to attest to the integrity of their research when submitting a manuscript for publication. To help prevent unjustified claims of authorship, this standardized form could also include the author’s statement that he or she contributed to the development of the manuscript. According to Hess and colleagues (2015), “Unjustified claims of authorship in scientific publications are referred to as a form of scientific misconduct. …[A]ppropriate authorship credit has become a decisive factor in the careers of young researchers and it needs to be managed and protected accordingly.” Ideally, the criteria qualifying an individual for authorship should be standardized by the scientific publication community. The Guidelines of the Vancouver Group [part of the International Committee of Medical Journal Editors (ICMJE)], which have been adopted by more than 600 biomedical journals to date, and those of the Committee on Publication Ethics (COPE) include criteria for appropriate assignment of authorship. Without this understanding, illegitimate exclusion of authors can occur, which does not allow one to understand fully who wrote or contributed to the work. Another aspect of the peer review process that should be standardized is the conflict of interest (COI) forms required by journals. Journals should collaborate to develop a single standardized COI form to be used with both authors and reviewers. ICMJE has initiated such an effort and offers the ICMJE Form for Disclosure of Potential Conflicts of Interest (International Committee of Medical Journal Editors 2017a). The Federal Demonstration Partnership (2018) has established a website to allow institutions and other entities to verify their compliance with COI forms.

There are guidelines and recommendations developed by other organizations that would also be useful in the development of a standardized form and can help to improve the peer review process overall. ICMJE developed the “Recommendations for the Conduct, Reporting, Editing and Publication of Scholarly Work in Medical Journals” to review best practice and ethical standards in the conduct and reporting of research and other material published in medical journals and to help authors, editors, and others involved in peer review and biomedical publishing create and distribute accurate, clear, reproducible, unbiased medical journal articles (International Committee of Medical Journal Editors 2017b). The Council of Science Editors (2012) published “CSE’s White Paper on Promoting Integrity in Scientific Journal Publications,” which aims to open dialogue about ethical publishing practices, inform those involved in the editorial process, and foster informed decision making by editors.

Peer review faces an ongoing challenge because of the limited number of individuals who are willing to serve as reviewers, which is further exacerbated by the exponential increase in papers needing review (Scientific Integrity Consortium meeting discussion, 2 March 2017). To address this gap, the Consortium suggested that serving as a reviewer should be a role that is built into career advancement. To a minor extent, this is already being implemented for tenure-earning faculty at some universities. This benefit provides an incentive for scientists to participate and further ensures that those who are experts in their field will be peer reviewers. The use of specific reviewers who review only certain parts of a paper that pertain to their expertise may also increase the quality of the review. Publishing the reviewers’ names yearly, as is currently practiced by some journals, may also increase the willingness of reviewers to evaluate papers.

More extensive training of peer reviewers is recommended so that they fully understand their duties. There is a need for the development of a reviewer manual or training guide that includes a list of specific tasks that are expected to be conducted by the peer reviewer. Part of this list of tasks should mirror the criteria that authors are asked to address when submitting a manuscript. One approach would be to develop a core training manual that individual journals or scientific disciplines could use and then incorporate supplementary material specific to their field of research. Training or resources geared toward graduate students, postdoctoral students, and young researchers would also develop a new generation of adept peer reviewers.

It is important to experiment with innovative review models that encourage transparency, which ultimately will increase scientific rigor. For example, the American Society for Microbiology is experimenting with a special review track called *m*-*Sphere Direct* within its open-access journal *m*-*Sphere*. Within this review track, authors work with reviewers directly and provide the editors the reviews they have obtained. The names of the reviewers are published together with the paper and, optionally, also the reviews. This experiment aims to shift the role of the reviewer from an anonymous critic hidden behind a curtain to a form of collaboration with the author. The goal is to further improve the quality and the rigor of the paper and the speed of publication, and in a more open and verifiable manner in which authors, reviewers, and editors are engaged in a transparent process.

Much of what is presented in this best practice also applies to procedures in grant reviews.

7. Encourage scientific journals to publish unanticipated findings that meet standards of quality and scientific integrity

By and large, tenure and promotions depend, in part, on an individual’s number of publications and the impact factor of the journals in which the papers are published. Most high-impact journals prefer to publish statistically significant and interesting results, which discourages scientists from submitting their less novel, negative, or null findings. Thus, the current research environment rewards the publication of positive results, and yet negative results and null findings are often just as important to advancing the scientific evidence base.

One of the consequences of this bias toward publication of statistically significant and interesting results is that human and financial resources could be dedicated to addressing the same, previously addressed research questions, because the null or negative results were not previously published. As research dollars are limited, the scientific community owes it to society to correct this practice. Additionally, unpublished negative results bias the body of work. Scientists should publish negative and null results, either in a journal or an online repository. A support system is needed to help and encourage scientists to publish such results and promote and reward the contribution of these findings. There has been some progress to address this issue. For example, the now defunct *Journal of Negative Results in Biomedicine* (*JNRBM*) (BioMed Central 2017) was an open-access, peer-reviewed journal that provided a platform for the publication and discussion of non-confirmatory “and negative” data, as well as unexpected, controversial, and provocative results in the context of current tenets. From its inception in September 2002, *JNRBM* provided a platform for results that would otherwise have remained unpublished, and many other journals (e.g., *PLoS One*, the *Frontiers* series, and *F1000*) followed *JNRBM*’s lead in publishing articles reporting negative or null results. *JNRBM s*ucceeded in its mission and ceased publication in September 2017, as it was claimed that there was no longer a need for a specific journal to host these null results (BioMed Central 2017).

Furthermore, the Consortium recommends that the current terms to describe results (“positive” and “negative”) should be replaced with “anticipated” and “unanticipated.” This simple change in terminology can transform the stigma surrounding the publication of unanticipated findings and encourage journals to publish them.

Registered Reports are another approach that is being instituted to encourage publication of all findings. According to Elsevier (2013), “Registered Reports are a form of empirical article in which the methods and proposed analyses are preregistered and peer reviewed prior to research being conducted. High quality protocols are then provisionally accepted for publication before data collection commences. This format of article is designed to reward best practices in adhering to the hypothetico-deductive model of the scientific method. It neutralizes a number of questionable research practices, including low statistical power, selective reporting of results, and publication bias, while also allowing complete flexibility to conduct exploratory (unregistered) analyses and report serendipitous findings.” Chris Chambers (2014) states the following:

The idea of accepting papers before results are known moves us beyond the assumption that the visibility of a scientific study should depend on its outcome… The reason for this publication bias is simple human nature: in judging whether a manuscript is worthy of publication, editors and reviewers are guided not only by the robustness of the method but by their impressions of what the results contribute to knowledge. Do the outcomes constitute a major advance, worthy of space within a journal that rejects the majority of submissions? Results that are novel and eye-catching are naturally seen as more attractive and competitive than those that are null or ambiguous, even when the methodologies that produce them are the same. This bias, in turn, creates perverse incentives for individuals. When we reward scientists for getting “publishable results”, we encourage a host of questionable practices to produce them.

The Center for Open Science (2018) is helping to lead the effort to make Registered Reports more commonplace. Their website states that currently 80 journals use the Registered Reports publishing format either as a regular submission option or as part of a single special issue. Other journals offer some features of the format (Center for Open Science 2018).

Full transparency of scientific findings is a critical component in the entire effort of trust in science and should be regarded as an ethical expectation. Scientists must be willing to disclose all findings, regardless of whether the findings support the research hypothesis, either in the peer-reviewed literature or in accessible online repositories. This is a key principle of integrity, because if it is not followed, the suppression of scientific findings can create a breach of trust in science and biased literature.

8. Seek harmonization and implementation among journals of rapid, consistent, and transparent processes for correction and/or retraction of published papers

Once a paper is published, it is the responsibility of both the author(s) and the journal to correct or retract it if an invalidating error or research misconduct is detected. Unfortunately, there are few or no incentives for journals and authors to go through the correction or retraction process and many disincentives. There are risks, such as defamation, breach of contract, or professional embarrassment, and the process of correcting or retracting papers varies widely across journals (Allison et al. 2016). However, in general, the benefits of correcting the record to the scientific community and society at large outweigh the risks. Many journals, with the help of organizations like COPE, are currently working to standardize and codify the language and processes of article corrections and retractions. Once these standards are finalized and adopted, they would ideally be used uniformly across all journals as best practices for the rapid, consistent, and transparent correction and retraction of papers. The Consortium recommended the development of new terminology for the retraction or correction process because of the range of reasons, from honest errors to confirmed research misconduct. The goal of this terminology change is to de-stigmatize corrections of honest errors.

Many underlying themes of this best practice are touched upon in Best Practices 6, 7, and 9.

9. Design rigorous and comprehensive evaluation criteria that recognize and reward the highest standards of integrity in scientific research

The Consortium encourages the scientific community to undertake the development of evaluation criteria and other ways of measuring integrity in scientific research, and to develop incentives and rewards that encourage scientific excellence and recognize outstanding work.

Buckwalter and colleagues (2015) stated that “Science, being a high-stakes enterprise, is based on the ability to produce new and important observations. An academic and/or industry scientific career is dependent on publication, which in turn has an impact on continued employment, promotion, grant support, personal recognition, and competition with other investigators.” Presently, there are metrics to evaluate an individual scientist, but they do not fully encompass the spirit and goal of this best practice. One existing way that a researcher’s career is evaluated is through the *h*-index, which “attempts to measure both the productivity and citation impact of the publications of a scientist or scholar” and is “based on the set of the scientist’s most cited papers and the number of citations that they have received in other publications” (Wikipedia 2017a). Although the *h*-index can be a valuable measurement, it may not sufficiently evaluate early career scientists who have fewer publications. The *h*-index also may not truly reflect how significant the research is and its true long-term impact to society.

The scientific community should search for additional and better metrics to evaluate scientists, such as mentoring and other non-publication–oriented activities, and to evaluate the value of their research. This best practice of evaluating and rewarding scientific integrity highlights the need for changes in promotion and tenure systems, including revising criteria for an individual’s professional review and advancement, such as evidence of training on issues of scientific integrity, a commitment to preregistered research plans and open science, the publication of unanticipated findings, responsibly and proactively correcting the research record, and contributing to the peer review process as a reviewer.

The Consortium proposes the development of metrics that support the measurement of the highest standards of scientific integrity in research for an individual and an institution. Once these metrics are created, the next step would be to conduct research on their efficacy to measure scientific integrity and whether they were successful in driving behavior that encourages scientific integrity. This could perhaps be taken on by the RIAB, as described in the *Fostering Integrity in Research* report, because one of the RIAB’s charges would be to “foster research integrity by stimulating efforts to assess research environments and to improve practices and standards” (National Academies of Sciences, Engineering, and Medicine 2017, p. 5). This new research on the efficacy of the developed metrics could be submitted to the World Conferences on Research Integrity Foundation (2017), which is developing a Research on Responsible Conduct of Research Registry. Creating universally acknowledged metrics that measure scientific integrity would drive adherence to scientific integrity more than any other single effort. The Consortium acknowledged that this is a bold concept that will not easily be undertaken but felt strongly that the scientific community needs to make a commitment to taking on this challenge.

## Summary

The Consortium believes that this set of recommended principles and best practices is broad and inclusive of the needed practices for instilling scientific integrity and can be used to better equip scientists to operate and be supported in a rapidly changing research environment. Traditional scientific integrity values in the research enterprise cannot be assumed to pass informally from one generation to the next but must be fostered to keep scientific integrity relevant. Science is a community built on trust; therefore, it is the responsibility of everyone to foster and promote a culture of scientific integrity.

All of the organizations from which the Consortium members were drawn have not yet formally endorsed or approved the principles and best practices but have offered to help disseminate them.

## Going forward

There must be community consensus and alignment around the necessity for scientific integrity standards and their content. This work is a step toward this goal of harmonizing principles and best practices across institutions and developing a standardized approach, along with effective tools for scientists, to achieve research accountability and integrity. The Consortium hopes these principles and best practices will help spark further dialogue and global conversations and partnerships that can take place moving forward.

The Consortium plans to develop a campaign, beginning in 2018, to raise visibility of these principles and best practices at professional society meetings and other venues. The goal of the campaign is to drive adoption of the principles and best practices and, ultimately, have a positive impact on the quality of science. As part of the campaign, the Consortium will be using the presentations as listening sessions to ask for feedback on the principles and best practices and how attendees will be able to put them into practice.

Moving forward, the Consortium will explore the development of the recommended checklist (outlined in principle 1) and the development of metrics to measure scientific integrity (outlined in Best Practice 9), potentially in collaboration with other organizations.

## List of Scientific Integrity Consortium Participants

- **Todd Abraham**, PhD, Formerly of the ILSI Global Board of Trustees.
- **David B. Allison**, PhD, Indiana University School of Public Health, Bloomington.
- **Georges Benjamin**, MD, American Public Health Association.
- **Stefano Bertuzzi**, PhD, MPH, American Society for Microbiology.
- **Kathryn J. Boor**, PhD, Cornell University.
- **John Coupland**, PhD, CFS, Institute of Food Technologists, Pennsylvania State University.
- **Johanna Dwyer**, DSc, RD, Tufts Medical Center.
- **Andrea Grantham**, Canadian Nutrition Society.
- **Francesca Grifo**, PhD, U.S. Environmental Protection Agency.
- **Linda J. Harris**, PhD, CFS, International Association for Food Protection, University of California, Davis.
- **Eric Hentges**, PhD, Formerly of ILSI North America.
- **Rachelle Hollander**, PhD, Formerly of The National Academies of Sciences, Engineering, and Medicine, The National Academy of Engineering, Center for Engineering Ethics and Society.
- **Chavonda Jacobs-Young**, PhD, U.S. Department of Agriculture, Agricultural Research Service.
- **Dennis Keefe**, PhD, U.S. Food and Drug Administration, Center for Food Safety and Applied Nutrition.
- **Alison Kim**, PhD, American Gastroenterological Association.
- **Alison Kretser**, MS, RD, ILSI North America.
- **Erin Landis**, American Gastroenterological Association.
- **Delia Murphy**, PMP, Formerly of ILSI North America.
- **Rosetta Newsome**, PhD, CFS, Institute of Food Technologists.
- **Sarah Ohlhorst**, MS, RD, American Society for Nutrition.
- **Maria Oria**, PhD, The National Academies of Sciences, Engineering, and Medicine, Health and Medicine Division, Food and Nutrition Board.
- **Kathryn Partin**, PhD, U.S. Department of Health and Human Services, Office of Research Integrity.
- **Sarah Rovito**, PE, Association of Public and Land-Grant Universities.
- **Nathan Sabel**, U.S. Food and Drug Administration, Office of Scientific Integrity.
- **Susan Sauer Sloan**, The National Academies of Sciences, Engineering, and Medicine, Government-University-Industry Research Roundtable.
- **Yvette Seger**, PhD, Federation of American Societies for Experimental Biology.
- **Alison Steiber**, PhD, RDN, Academy of Nutrition and Dietetics.
- **Kimberly Stitzel**, MS, RD, American Heart Association.
- **Dorothea Vafiadis**, MS, FAHA, Formerly of the American Heart Association.
- **Karen Wallace**, Secretariat on Responsible Conduct of Research (Canada), Representative for the Canadian Institutes of Health Research, the Natural Sciences and Engineering Research Council of Canada, and the Social Sciences and Humanities Research Council of Canada.
- **Catherine Woteki**, PhD, Formerly of the U.S. Department of Agriculture, Research, Education, and Economics.
- **Jessica Wyndham**, American Association for the Advancement of Science.
- **Rickey Yada**, PhD, University of British Columbia.

## Footnotes

1 The term “p-hacking,” also known as inflation bias or “selective reporting,” refers to the misreporting of true effect sizes in published studies. It occurs when researchers try out several statistical analyses and/or data eligibility specifications and then selectively report those that produce significant results (Head et al. 2015).
